# Profiling, isolation and characterisation of beneficial microbes from the seed microbiomes of drought tolerant wheat

**DOI:** 10.1038/s41598-021-91351-8

**Published:** 2021-06-07

**Authors:** Holly Hone, Ross Mann, Guodong Yang, Jatinder Kaur, Ian Tannenbaum, Tongda Li, German Spangenberg, Timothy Sawbridge

**Affiliations:** 1 Agriculture Victoria Research, Department of Jobs, Precincts and Regions, AgriBio, Centre for AgriBioscience, Bundoora, VIC Australia; 2 School of Applied Systems Biology, La Trobe University, Bundoora, VIC Australia; 3 College of Animal Science and Technology, Henan University of Science and Technology, Luoyang, Henan People’s Republic of China; 4 Agriculture Victoria Research, Department of Jobs, Precincts and Regions, AgriBio, Centre for AgriBioscience, 5 Ring Road, Bundoora, VIC 3083 Australia

**Keywords:** Metagenomics, Microbiome

## Abstract

Climate change is predicted to increase the incidence and severity of drought conditions, posing a significant challenge for agriculture globally. Plant microbiomes have been demonstrated to aid crop species in the mitigation of drought stress. The study investigated the differences between the seed microbiomes of drought tolerant and drought susceptible wheat lines. Furthermore, it highlighted and quantified the degree of drought tolerance conferred by specific microbes isolated from drought tolerant wheat seed microbiomes. Metagenomic and culture-based methods were used to profile and characterise the seed microbiome composition of drought tolerant and drought susceptible wheat lines under rainfed and drought conditions. Isolates from certain genera were enriched by drought tolerant wheat lines when placed under drought stress. Wheat inoculated with isolates from these targeted genera, such as *Curtobacterium flaccumfaciens* (*Cf* D3-25) and *Arthrobacter* sp. (*Ar* sp. D4-14) demonstrated the ability to promote growth under drought conditions. This study indicates seed microbiomes from genetically distinct wheat lines enrich for beneficial bacteria in ways that are both line-specific and responsive to environmental stress. As such, seed from stress-phenotyped lines represent an invaluable resource for the identification of beneficial microbes with plant growth promoting activity that could improve commercial crop production.

## Introduction

Drought poses significant challenges to agricultural production in arid and semi-arid regions of the globe. Increases in mean global temperatures and decreased frequency and intensity of precipitation, as a result of climate change, are extending periods of drought and decreasing crop yields^1^. These shifts highlight the increasing need for abiotic stress tolerance in crop species^1,2^. *Triticum aestivum* (wheat) is grown on approximately 220 million hectares of land globally, producing 20% of the world’s caloric requirements^3^. In Australia, drought conditions have had severe impacts on wheat production, reducing the 2019 winter harvest to 35% below the 10 year average^4,5^. Research into mechanisms to increase drought tolerance in crop plants has focused primarily on improving crop genetics using techniques such as QTL mapping, marker assisted breeding and introgression from wild species^6–9^. However, the identification of drought tolerance with high heritability is confounded by complex genotype by environment interactions^7–10^. Analysis of arid and drought envirotypes has identified microbiome composition as an environmental factor that affects drought tolerance in crop plants^11,12^. Envirotyping, a term coined by Cooper et al., is a comprehensive method of measuring environmental factors with the capacity to affect phenotypic variation in plant growth designed to aid genotype by environment (GEI) and phenotype prediction^11,13^. By examining wheat microbiomes from arid and drought environments, it is possible to identify microbial agents that augment the phenotype conferred by the genome of the host plant and hence confer drought tolerance. As a result, there is increasing interest in interrogating the plant microbiome with the goal of identifying microbes that can increase drought tolerance in tandem with more traditional breeding methods^14–18^.

Plants form intimate relationships with microbes that colonise their tissues and organs, forming a single ecological unit known as a holobiont^19^. These microbiomes have been demonstrated to influence a myriad of plant traits such as germination, biomass accumulation, pathogen resistance and abiotic stress tolerance^20^. Seeds harbor the initial inoculum of microbes, playing a vital role in the transmission of microbial resources between plant generations^21^. As the initial colonisers of plant tissue, the seed microbiome is believed to shape the overall composition of the plant microbiome and therefore have a competitive advantage over microbes recruited from soil and root^22,23^. It is postulated that the seed microbiome evolves as a result of host selection for traits that complement the plant genome^19,24^. While root microbiomes have been the focus of significant study, the seed microbiome has largely been overlooked^25^. Despite the unique relationship between seed microbiomes and host, and the particular advantage this relationship could confer, a comparatively limited number of bacterial genera have been detected in seed microbiomes compared to other plant microbiomes^24^.

Studies into the influence of drought on plant microbiomes have focused almost exclusively on root microbiomes^12,17,26,27^. Several gram-positive rhizosphere bacteria have been demonstrated to be effective in improving drought stress tolerance in crop plants^28–30^. Timmusk et al., demonstrated that wheat treated with *Paenibacillus* and *Bacillus* species isolated from arid environments increased plant biomass by up to 78% and increased the plant survival rate fivefold under severe drought conditions^31^. Notably, *Paenibacillus* and *Bacillus* species isolated from moderate drought environments did not increase drought tolerance in wheat^31,32^. The consensus amongst many plant microbiome studies is that drought conditions have a significant impact on the composition and diversity of the microbiome, often leading to a marked increase in *Actinobacteria* in root microbiomes^12,17,27^. It is clear from these studies that recruitment and enrichment of certain bacterial elements in root microbiomes were driven by host-specific metabolic and phenotypic factors^12,27^. Further to this, microbiomes have also shown line-specific enrichment in root microbiomes of rapeseed, cannabis and wheat^15,18,33^.

We hypothesise that seed microbiomes from plants exposed to abiotic stress enrich for bacteria that are more tolerant of abiotic stress, and that the composition of seed microbiomes differ between drought susceptible and drought tolerant wheat lines.

In this study, we present the first analysis of seed microbiomes from contrasting drought tolerant and drought susceptible wheat lines under drought and rainfed conditions, with the aim of identifying microbes that are capable of increasing drought tolerance in a host plant. By interrogating the seed microbiome in silico, endophytes and epiphytes can be identified and isolated that have the potential to increase the drought tolerance of crop species at a commercial level.

## Methods

### Seed source

Seeds from seven lines of *Triticum aestivum* were sourced from the Grains Innovation Park in Horsham, Victoria, Australia. These seeds were harvested in 2017 from a field trial where 43 wheat lines were examined for drought response. Each line was subjected to rainfed and drought conditions, simulated using rainout shelters. Each of the 43 lines were planted in three replicates per treatment, under the same soil conditions in a randomised design. The drought susceptibility index (DSI) was calculated for each line, using yield differences under drought and rainfed conditions. The lines were subsequently classed as either drought tolerant, or drought susceptible. A subset of seven lines were used in this study and represented the four most drought tolerant and three most drought susceptible lines (Supplementary Table 1).

### Culturing the seed microbiome

#### Microbial isolation

For microbial isolation, a subset of six lines were chosen from the seven lines. Bacteria were isolated from the seeds of four drought tolerant wheat lines (lines 1, 2, 3 and 4) and two drought susceptible wheat lines (lines 6 and 7). Ten seeds from each treatment were plated onto stacked pieces of sterile filter paper soaked in Nystatin (50 mg L^−1^). These seeds were germinated in the dark for 2 days, then grown for a further 4 days under light conditions. The seedlings were harvested and seed husks discarded. Plant tissues from the pooled seedlings were then immersed in phosphate buffered saline (PBS) and ground using a QIAGEN Tissuelyser Mixermill for up to one minute at 30 Hertz. A 10µL aliquot of the resulting macerate was added to 90 µl of PBS. Further 1:10 dilutions were performed to generate 10^–3^ and 10^–4^ solutions. Reasoners 2 Agar (R2A, Oxoid, Australia) agar were then inoculated with 10^–3^ and 10^–4^ solutions from each treatment and allowed to grow for 24–48 h. Individual bacterial colonies were then streaked onto single R2A plates to isolate and purify single bacterial strains. A total of 438 bacterial strains were isolated from the six wheat lines and stored in 20% glycerol at − 80 °C.

#### Microbe identification using MALDI-TOF

The microbial isolates were putatively identified using the Bruker MALDI Biotyper system. The bacterial strains were taken from the − 80 °C glycerol stock, plated onto R2A and grown for 48 h. Single colonies were taken from the isolate plates and prepared for analysis using the manufacturer’s Extended Direct Transfer method. The plate was analysed using the Bruker MALDI-TOF ultrafleXtreme in accordance with the manufacturer’s instructions. *Escherichia coli* strain ATCC 25922 was used as a quality control and as an internal standard. The resulting protein spectra were processed using the MALDI BioTyper automation 2.0 software at default settings. The microbes were then assigned a preliminary identification by comparing raw protein spectra against known spectra in the MALDI BioTyper library, which contained 2750 species from 471 genera as of January 2019. The Biotyper library was supplemented with an in-house database generated from previous endophyte studies of this research group. The protein spectra were processed using an in-house Refiner pipeline (GeneData 13.5). A hierarchical clustering algorithm in Analyst (GeneData 13.5) was used to create a phenogram that grouped bacterial strains based on the similarity of protein profiles.

### Microbiome profiling

#### 16S rRNA library preparation and sequencing

The microbiomes of the four drought tolerant lines (lines 1, 2, 3 and 4) and three drought susceptible lines (lines 5, 6, and 7) were profiled. Seeds from each line were plated onto filter paper soaked with sterile water and germinated in the dark at room temperature for 2 days. The seedlings were grown for a further 4 days under light conditions. Seedlings of approximately equal size were harvested and the seed husks discarded. Ten replicates, consisting of the plant tissues from five pooled seedlings, were used for each line. Each replicate was snap frozen in liquid nitrogen. DNA was then extracted from each sample. Minor modifications were made to the QIAGEN MagAttract 96 DNA Plant Core Kit during DNA extraction to allow for use of the Biomek FX liquid handling station. The V4 hyper variable region of the 16S rRNA gene was targeted for microbiome profiling using the Illumina 16S Metagenomic Sequencing Library Preparation protocol (Methods S2) in conjunction with PNA PCR blockers to reduce amplification from plant organelles^34^. Paired-end sequencing was performed on an Illumina HiSeq3000 using the 2 × 150 bp v3 chemistry cartridge.

#### Bioinformatics

Microbiome bioinformatics were performed with a combination of PANDAseq and QIIME2 2019.10. The paired-end Illumina reads were trimmed and assembled using PANDAseq^35^. Using QIIME2 (v 2019.10), the 16S sequences were filtered and denoised using Deblur (via q2-deblur)^36,37^. There was a total of 18,220,959 quality-filtered reads Taxonomy was assigned to OTUs using SILVA132 99% v4 (via q2-feature-classifier)^38,39^. All amplicon sequence variants (OTUs) were aligned using MAFFT (via q2-alignment) and a phylogeny was created using fasttree (via q2-phylogeny)^40,41^. Alpha-diversity metrics were analysed such as observed features and Shannon’s phylogenetic diversity, and beta-diversity metrics such as Robust Aitchison PCA^42^. The rarefaction curve reached an asymptote, indicating that sampling had captured the diversity of the seed microbiomes. To capture the diversity of the microbiomes, a sampling depth of 15,000 reads was applied across samples. Analysis and visualisation were performed using QIIME2 pipelines. The QIIME2 feature table was exported in biom format and ANOVA analysis performed on individual OTUs^43^.

### Plant growth promotion effects of the cultured microbiome

#### Evaluation of microbiome bacteria for biostimulation of *Triticeae*

A representative isolate of two dominant cultured genera (*Curtobacterium flaccumfaciens*—*Cf* D3-25; *Arthrobacter* sp. *Ar* sp. D4-14) from drought tolerant lines were selected and assessed for a biostimulation effect in *Triticeae*. These genera were also significantly higher in either drought tolerant lines compared to drought susceptible (*Cf* D3-25) or drought conditions compared to rainfed conditions (*Ar* sp. D4-14) in the microbiome profiling analysis. Bacterial strains *Cf* D3-25 and *Ar.* sp D4-14 were cultured in Lysogeny Broth (LB, Oxoid, Australia) overnight at 26 °C. After 24 h (h), seeds of *Triticum aestivum* (cv Bob White Red Haplotype) were sterilised by soaking in 80% ethanol for 3 min (min), then washed five times in sterile distilled water. The cultures were centrifuged and washed in PBS twice before being resuspended in their original volume of overnight culture. These cultures were then diluted step-wise with PBS to concentrations of 10^–1^, 10^–2^, 10^–3^, 10^–4^ and 10^–5^ respectively. Seeds were soaked in undiluted, or diluted solutions for four h at 26 °C in a shaking incubator. As a control, seeds were soaked in PBS without bacteria under the same conditions. Fifteen inoculated seeds were then placed on moist sterile filter paper in sterile Petri plates and allowed to grow for 7 days at room temperature. There were four replicates per treatment. To measure the root length, the seedlings were removed from the filter paper and the longest root was measured. The same assay was performed using ryecorn, barley (cv Hindmarsh), oat, and spelt (cv. ST1040) using the methods described above. Data was statistically analysed using a one-way ANOVA and Tukey test to determine any significant difference (p ≤ 0.05) between treatments using OriginPro 2018 (v b9.5.1.195).

#### Evaluation of microbiome bacteria for drought mitigation in wheat

Wheat seeds (cv Bob White Red Haplotype) were sterilised according to the method described in section “Evaluation of microbiome bacteria for biostimulation of Triticeae”. The seeds were then soaked in overnight cultures of either *Cf* D3-25, or *Ar.* Sp for 4 h at 26 °C in a shaking incubator. For control seedlings, seeds were soaked in sterile LB for 4 h at 26 °C in a shaking incubator. These seeds were planted in a glasshouse in a potting medium containing a mixture of 25% Biogrow potting mix, 37.5% Fertool vermiculite and 37.5% Fertool perlite. For each treatment, eight seeds were planted at a depth of one centimetre (cm) around the edge of each pot (200 m W × 190 mm H, Garden City Plastics) for a total of 12 pots per treatment. An initial experiment was conducted to determine the appropriate watering regimes to induce moderate and severe drought conditions. During this experiment, plants administered with 150 mL and 50 mL showed clear phenotypic differences from the control well-watered (300 mL) treatments in shoot weight, root weight, number of leaves and shoot length. A repeat experiment was conducted to statistically validate the effects of the bacteria in mitigating drought. During the two experiments, all pots were well-watered to ensure even germination. After the first week of growth (BBCH scale 11), seeds that had not germinated were removed, reducing the total number of plants per pot to four. The bacterial-treated seeds were subjected to one of the three watering conditions. Pots under the well-watered, mild drought, or severe drought condition received 300 mL, 150 mL, or 50 mL of water respectively, every 48 h. After 6 weeks of growth (BBCH scale 22–25), the plants were harvested and washed to remove soil debris. The shoot and root tissue were measured with respect to length and weight. This data was analysed using OriginPro 2018 (Version b9.5.1.195) as described in “Evaluation of microbiome bacteria for biostimulation of Triticeae”.

## Results

### Culturing the seed microbiome

A total of 438 bacteria strains were isolated from the seed microbiomes of six wheat lines—four drought tolerant and two drought susceptible lines. As the focus of the study was on the interrogation of resources available in the microbiome of drought tolerant wheat lines, only two drought susceptible lines were analysed to allow comparisons to be drawn between culturable microbiomes. At a phylum level, the isolates were identified as either *Gammaproteobacteria* (42.9%), *Actinobacteria* (27.2%), or *Firmicutes* (0.2%). At a genera level, the isolates belonged overwhelmingly to four genera, *Pantoea* (25.3%), *Pseudomonas* (17.4%), *Arthrobacter* (12.8%) and *Curtobacterium* (12.6%), while minor genera consisted of *Rathayibacter* (0.5%), *Clavibacter* (1.1%), *Erwinia* (0.2%) and *Paenibacillus* (0.2%). Unidentified isolates comprised 29.9% of total isolates.

**Figure 1:**
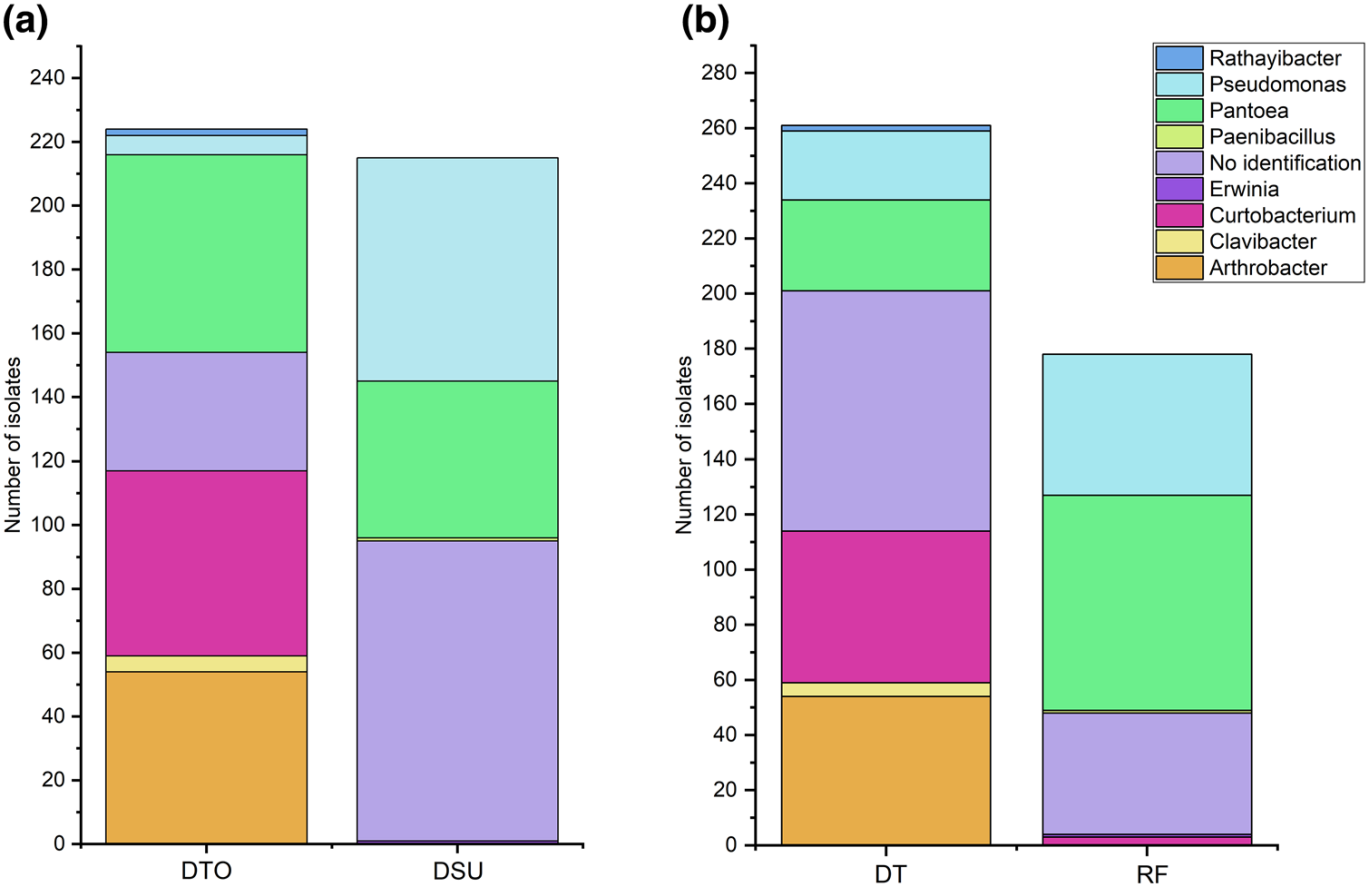
Bacteria isolated from the seeds (**a**) of drought tolerant lines (DTO) and drought susceptible lines (DSU) and (**b**) from wheat under either drought (DT) and rainfed conditions (RF).

*Pantoea* and *Pseudomonas* were identified in both drought susceptible lines (DSU) and drought tolerant lines (DTO) (Fig. 1). *Pantoea* and *Pseudomonas* were the dominant genera in DSU comprising 32.6% and 22.8% of isolates respectively, as opposed to 27.6% and 2.7% of isolates in DTO. *Pantoea* and *Pseudomonas* were more prevalent under rainfed conditions (RF) comprising 43.8% and 26.8% of isolates, respectively, rather than 12.6% and 9.5% of isolates from drought conditions (DT).

*Arthrobacter* and *Curtobacterium* isolates were exclusive to DTO and were the dominant genera accounting for 24.0% and 25.8% of isolates, respectively. *Arthrobacter* isolates were also exclusive to DT, while *Curtobacterium* isolates dominated under DT (22.0%) as opposed to RF (1.7%). Furthermore, *Curtobacterium* was the only bacteria isolated from seeds of DTO line 3 under DT, while *Arthrobacter* was the only bacteria isolated from DTO line 4 under DT (Supplementary Table 2). *Clavibacter* and *Rathayibacter* were exclusively isolated from DTO under DT but only accounted for 2.2% and 0.9%, respectively. *Erwinia* and *Paenibacillus* were exclusive to DSU under RF, but only accounted for 0.5% and 0.5% of the population, respectively. Bacteria that were unable to be identified using MALDI-TOF were isolated from both DTO and DSU, accounting for 16.5% and 43.7% of isolates, respectively. Unknown isolates were also identified from DT and RF, accounting for 33.2% and 24.7% of isolates, respectively.

### Microbiome profiling

#### Variation in microbial diversity

**Figure 2:**
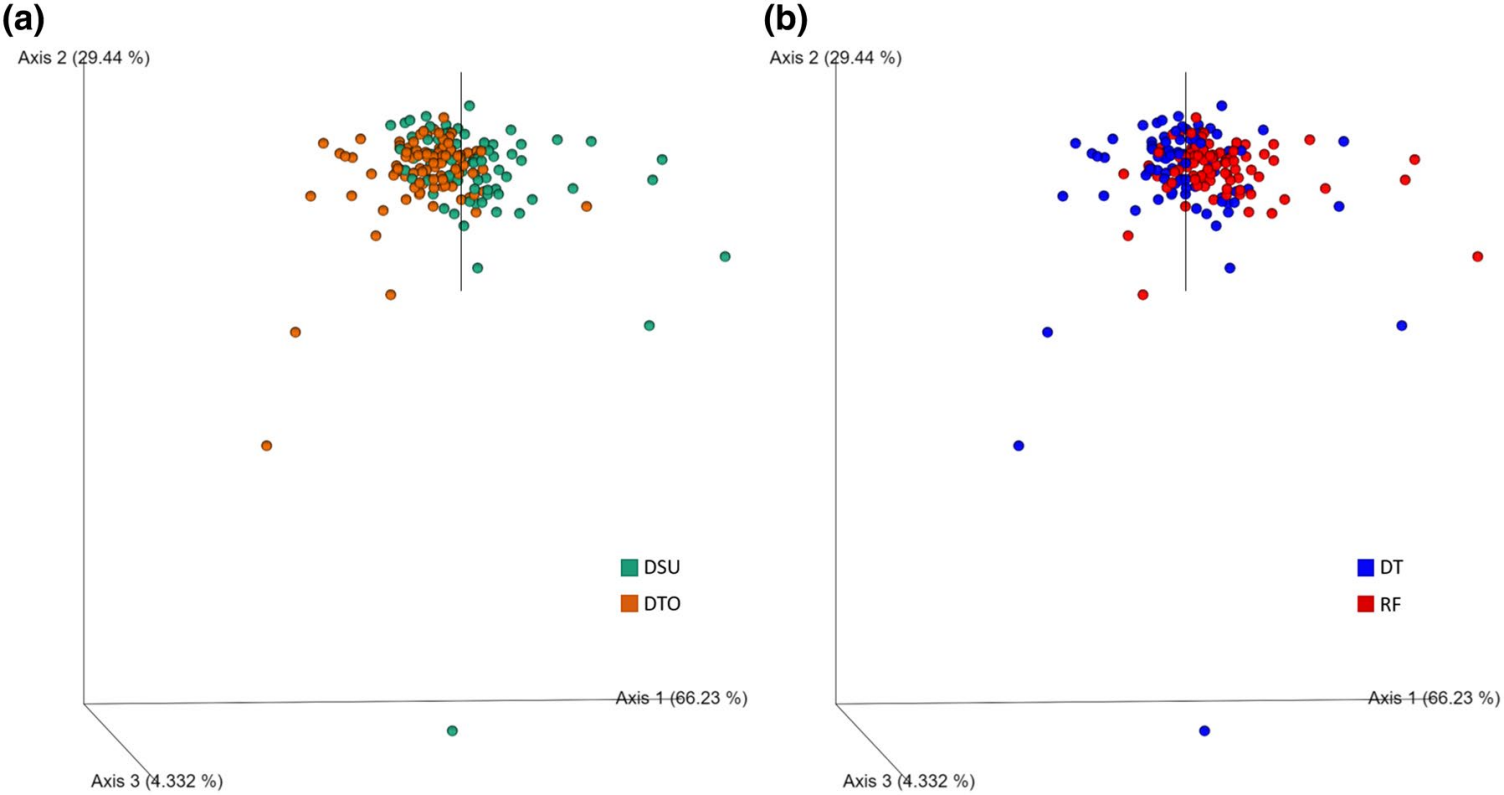
RPCA of the seed microbiomes of wheat lines (**a**) subjected to drought conditions (DT) or rainfed conditions (RF) (PERMANOVA pseudo-*F* = 21.0, *p* = 0.001) (**b**) from drought tolerant lines (DTO) or drought susceptible lines (DSU) (PERMANOVA pseudo-*F* = 8.8, *p* = 0.001).

The microbiomes of the seeds from DTO and DSU that had been subject to DT and RF were profiled using 16S rRNA amplicon sequencing—four from DTO and three from DSU. To characterise the microbial diversity of the wheat seed microbiomes, alpha and beta diversity analyses were performed. For alpha diversity, Shannon index comparison of DT microbiomes (8.8) was significantly more diverse than RF microbiomes (7.0) (q = 2.5e−2, H = 76.4, Kruskal–Wallis test). For beta diversity, Robust Aitchison PCA (RPCA) identified significant separation of the microbiome profiles of DTO from DSU (Fig. 2a, PERMANOVA pseudo-*F* = 21.0, *p* = 0.001). Similarly, there was significant separation between the microbiomes of wheat seeds of DC from RC (Fig. 2b, PERMANOVA pseudo-F = 8.8, p = 0.001).

#### Bacterial taxonomic composition

**Figure 3:**
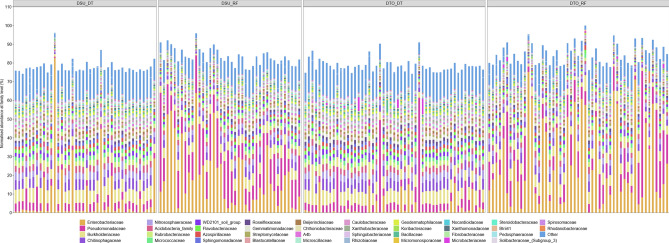
Comparison of the microbiomes (normalised percent abundance) of wheat plants under drought and rainfed conditions (DSU-DT—drought susceptible lines under drought conditions, DTO—DT drought tolerant lines under drought conditions, DSU-RF—drought susceptible under rainfed conditions and DTO-RF—drought tolerant under rainfed conditions) at the family level. Each column represents a microbial profile of a wheat line replicate under the above-mentioned specified conditions.

The composition of seed microbiomes was influenced by environmental conditions and wheat lines (Fig. 3), with the environmental conditions appearing to have the greatest impact. The abundance of the three most dominant families reducing substantially between DT and RF. For instance, in DTO under RF, the most dominant families were *Enterobacteriaceae, Pseudomonadaceae* and *Burkholderiaceae*, which represented 31.6%, 13.6% and 6.0% of isolates respectively, as opposed to 3.5%, 8.8% and 9.0% of isolates in DTO under DT. In DSU under RF, the most dominant families were *Enterobacteriaceae*, *Pseudomonadaceae* and *Burkholderiaceae*, which represented 23.3%, 19.7% and 6.0% of isolates respectively, as opposed to 10.7%, 5.7% and 8.5% of isolates in DSU under DT. In DTL under DC, the three most abundant families were *Burkholderiaceae* (31.6%), *Pseudomonadeceae* (13.6%) and *Chitinophagaceae* (6.0%). In DSL under DC, the three most abundant families were *Enterobacteriaceae (9.0% 31.6%)*, *Burkholderiaceae (8.8%)* and *Chitinophagaceae (6.7%)*.

Assessing OTU abundance between wheat lines (DTO and DSU) and environmental conditions (DT and RF) it was evident that certain OTUs were enriched in seed microbiomes under DT. ANOVA analysis of the microbiomes under DT and microbiomes under RF identified 1069 OTUs that were significantly different (p > 0.05) (Supplementary Table 3). The ten most significant OTUs belonged to the genera *Pseudomonas* (p = 9.0E−18), Unknown (ANOVA p = 2.1E−15, p = 1.7E-12, p = 1.6E−11), *Flavobacterium* (ANOVA p = 7.7E−13), *Amycolatopsis* (ANOVA p = 3.0E−12), *Bradyrhizobium* (ANOVA p = 3.7E−11), *Pantoea* (ANOVA p = 5.8E−11), *Skermenella* (ANOVA p = 6.0E−11) and *Rubrobacter* (ANOVA p = 7.5E−11). There were multiple significant OTUs that belonged to genera isolated from wheat seeds, including *Pseudomonas*, *Pantoea* and *Arthrobacter* that appeared in the top 0.2% of significant OTUs. In DTO Line 3 and DTO Line 4, *Arthrobacter* was enriched 1.5-fold (Tukey test, p = 2.2E−4) and 1.9-fold (Tukey test, p = 3.4E−6), respectively. Approximately a third of OTUs identified were either unknown at the genus level or uncultured at the genus level. Of the top 50 OTUs, 17 belonged to unknown or uncultured genera.

**Figure 4:**
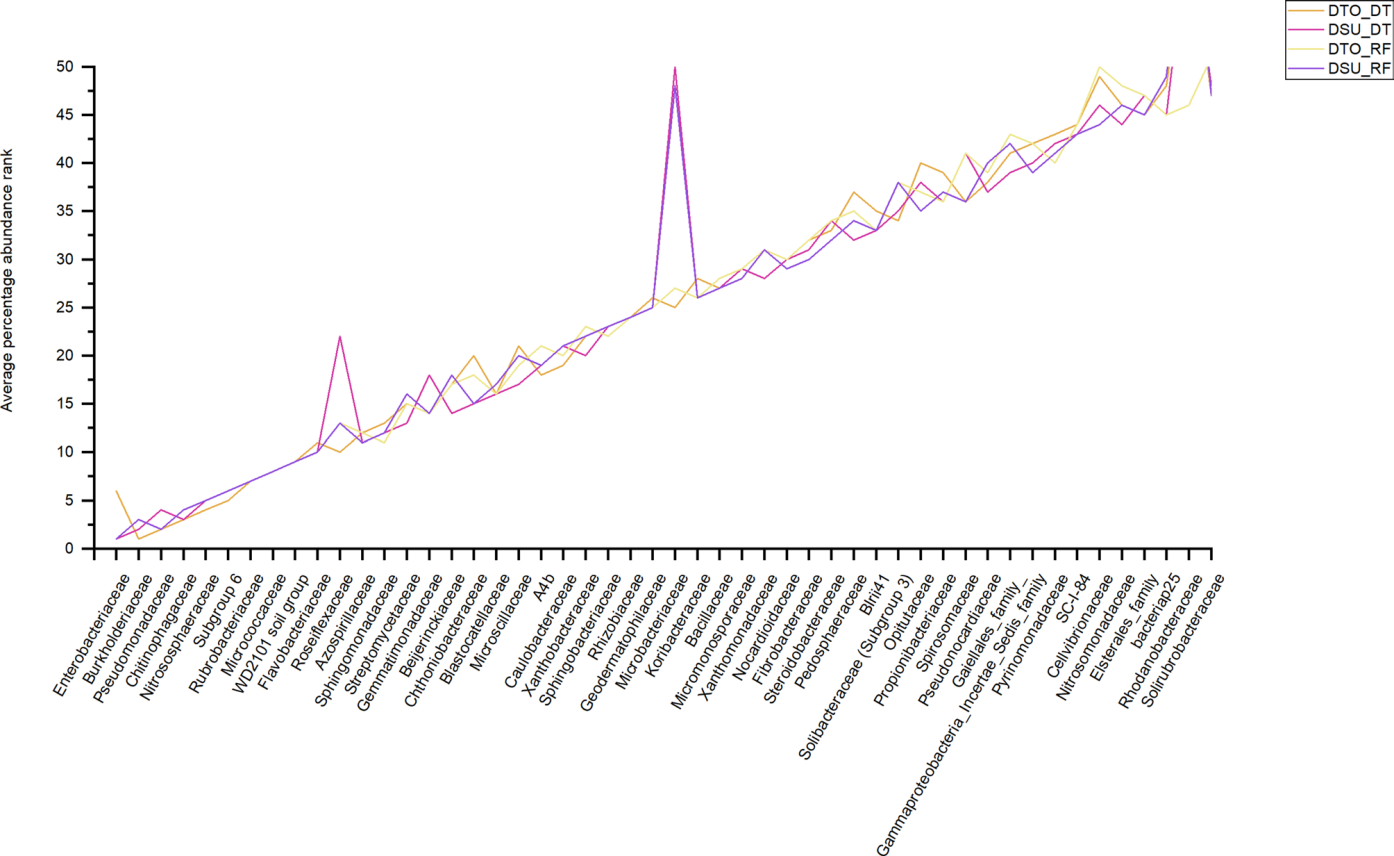
Comparison of the average percentage abundance of bacterial families between the two wheat lines (DTO and DSU), under the two environmental conditions (DT and RF). Families were sorted from highest to lowest based on percent abundance and ranked from highest value (Rank 1) to 50th value (Rank 50) for wheat line (DTO and DSU) and environmental condition (DT and RF). The minimum rank value was taken across the four lines/conditions, and the columns sorted from lowest rank to highest rank, with the rankings plotted as a line graph.

The microbiomes of DTO showed enrichment of certain microbes when compared with microbiomes of DSU, under both DT and RF (Fig. 3). For instance, at a family level the greatest differential was observed in the *Enterobacteriaceae* (see above) and *Microbacteriaceae*. DTO had a higher abundance of *Microbacteriaceae* (1.0%) compared to DSU (0.4%) under DT, while DTO also had higher abundance of Microbacteriaceae (0.5%) compared to DSU (0.3%) under RF. When ranking the relative abundance of OTUs at a family level, *Microbacteriaceae* were ranked at 25 for DTO under DT and 27 under RF (Fig. 4). Comparatively, *Microbacteriaceae* were ranked at 50 for DSU under DT and 48 under RF. Genera from the family *Microbacteriaceae* were significant in an ANOVA of the microbiome of DTO and DSU under DT, namely *Curtobacterium* (p = 3.3E−3), *Agromyces* (p = 0.03) and an unknown genera (p = 0.05) (Supplementary Table 4).

### Plant growth promotion effects of the cultured microbiome

#### Evaluation of microbiome bacteria for biostimulation of *Triticeae*

**Figure 5:**
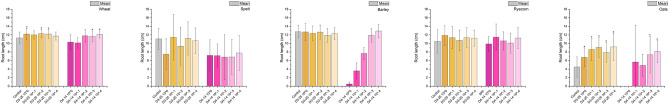
Biostimulation effects of *Cf* D3-25 or *Ar*. sp D4-14 in roots of Triticeae species. Root length (cm) of wheat, barley, oat, ryecorn and spelt seedlings inoculated with different concentrations (10^0^, 10^–1^, 10^–2^, 10^–3^ and 10^–4^) of *Cf* D3-25 or *Ar*. sp D4-14 (ANOVA, P < 0.05). Stars indicate inoculum concentrations that had significantly longer roots. Error bars show standard deviation.

To assess the growth promotion effect of *Cf* D3-25 and *Ar*. sp D4-14 on related crop species, a seedling assay was established with members of the tribe *Triticeae* (wheat—*Triticum aestivum*; spelt—*Triticum spelta*, durum—*Triticum durum*; ryecorn—*Secale cereale*; oats—*Avena sativa*; barley—*Hordeum vulgare*). Wheat seeds inoculated with different concentrations of *Cf* D3-25 or *Ar*. sp D4-14 were germinated and allowed to grow for 7 days to evaluate potential biostimulation activity. Overall, there was a root lengthening effect observed in microbe-treated wheat seedlings (Fig. 5). In early wheat seedlings inoculated in *Cf* D3-25 solutions diluted to 10^0^, 10^–2^ and 10^–3^ (containing 7 × 10^8^, 7 × 10^6^ and 7 × 10^5^ CFU mL^−1^, respectively), the root length was significantly longer compared to the uninoculated control, increasing root length by 7.9%, 9.1% and 8.0%. Similarly, in wheat seedlings inoculated with *Ar*. sp D4-14 solutions diluted to 10^–3^ and 10^–4^ (containing 1.13 × 10^6^ and 1.13 × 10^5^ CFU mL^−1^), the root length was significantly longer compared to the uninoculated control, increasing root length by 21.9% and 21.8%. Oat seedlings inoculated in *Cf* D3-25 solutions of 10^–1^, 10^–2^, 10^–3^ and 10^–4^ (8.2 × 10^7^, 8.2 × 10^6^, 8.2 × 10^5^, 8.2 × 10^4^ CFU mL^−1^, respectively) had significantly longer roots than the control, with a percentage increase of 90.8%, 101.6%, 63.9% and 104.7% respectively. Similarly, oat seedlings inoculated with *Ar*. sp D4-14 solutions of 10^–3^ and 10^–4^ (5.8 × 10^5^ and 5.8 × 10^4^ CFU mL^−1^, respectively) had significantly longer roots than the control, with a percentage increase of 63.9% and 80.1% respectively. There were no significantly longer roots observed in barley, spelt, or ryecorn when treated with either bacterial strain. Interestingly, high concentrations of *Ar*. sp D4-14 inhibited root growth in both barley and oats. There were no significant observable shoot effects in any *Triticeae* (Supplementary Figure 3).

### Evaluation of microbiome bacteria for drought mitigation in wheat

To assess the ability of *Cf* D3-25 and *Ar* sp. D4-14 to aid drought tolerance in wheat, an *in planta* experiment was established to assess growth under a range of drought conditions. At the end of 6-weeks of growth under well-watered (control), mild drought and severe drought conditions, the wheat plants were harvested, and shoot and root measurements were taken. Wheat plants inoculated with *Cf* D3-25 and *Ar*. sp D4-14 had significant increases in a range of shoot and root measurements compared to control (Tukey test), across a range of conditions.

**Figure 6:**
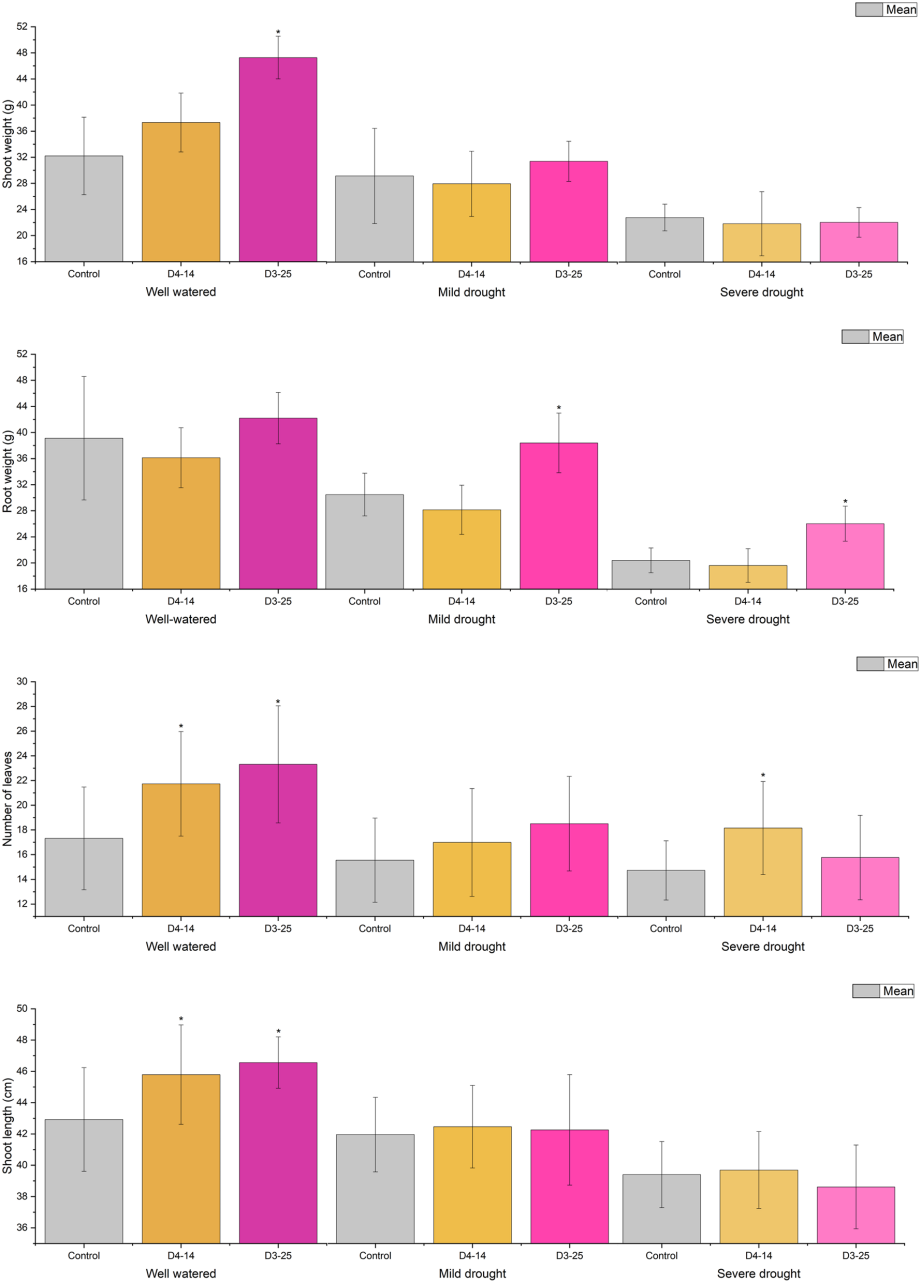
Growth of wheat plants inoculated with *Cf* D3-25 (pink) and *Ar* sp. D4-14 (orange) and control (grey), grown under well-watered (300 mL every 48 h), mild drought (150 mL every 48 h) and severe drought conditions (50 mL every 48 h)—(**a**) wet shoot weight and (**b**) wet root weight (**c**) Number of leaves and (**d**) shoot lengths. Error bars show standard deviation. Stars indicate bacterial treatments that were significantly difference from control.

For *Cf* D3-25-inoculated wheat plants, shoot weights were increased under well-watered (46.8% increase, p = 0.003) and mild drought (7.7% increase, p = ns) conditions compared to the control (Fig. 6a). Root weights were also increased in *Cf* D3-25-inoculated wheat under well-watered (7.8%% increase, p = ns), mild drought (26.0% increase, p = 0.04) and severe drought (27.6% increase, p = 0.04) conditions compared to the control (Fig. 6b). The number of leaves was greater in *Cf* D3-25-inoculated wheat under well-watered (34.7% increase, p = 0.001) and mild drought conditions (18.0%% increase, p = ns) compared to control (Fig. 6c). Finally, shoot lengths were increased in *Cf* D3-25-inoculated wheat under well-watered (8.5% increase, p = 0.002) compared to control (Fig. 6d).

For *Ar* sp. D4-14-inoculated wheat plants, shoot weights were increased under well-watered (16.0% increase, p = ns) conditions compared to the control (Fig. 6a). Root weights were not increased in *Ar* sp. D4-14-inoculated wheat under any condition compared to the control (Fig. 6b). The number of leaves was greater in *Ar* sp. D4-14-inoculated wheat under well-watered (25.5% increase, p = 0.02), mild drought (8.8% increase, p = ns) and severe drought (22.0% increase, p = 0.02) conditions compared to control (Fig. 6c). Finally, shoot lengths were increased in *Ar* sp. D4-14-inoculated wheat under well-watered (6.7% increase, p = 0.02) compared to control (Fig. 6d).

## Discussion

A combination of culture-based and metagenomic methods were used to characterize the seed microbiome composition of drought tolerant and drought susceptible wheat lines under rainfed and drought conditions. Variation in microbial abundance and diversity between seed microbiomes of these lines correlated with changes in water availability and plant host genetics. From the collection of cultured microbes, genera that had been enriched in seed microbiomes under drought conditions in drought tolerant lines were identified as potential plant growth promoting bacteria (PGPB). Subsequent screening revealed biostimulation effects and increased drought tolerance in wheat and related crop species inoculated with seed microbiome strains.

### Drought stress and plant host genetics shape the wheat seed microbiome

The seed microbiomes under DT had a higher Shannon’s index than their rainfed counterparts, indicating higher levels of diversity. There is conflicting data on the effects of drought on microbial diversity. In a study by Jochum et al., multiple generations of drought stress on wheat microbiomes lead to an increase in alpha and beta diversity^26^. However, in other studies the microbiomes of sorghum and wheat exhibited a marked decrease in diversity^27,44^. It has been suggested that increased diversity could be explained by selective enrichment for functionality, rather than taxonomy^26^.

As previously reported in studies on the root microbiomes of wheat, and more than 30 other genetically divergent plant species, there was a marked increase in *Actinobacteria* in the microbiomes of genetically distinct wheat lines when subjected to drought stress^12,17,26,44,45^. In this study, microbiome profiling showed *Actinobacteria* families (*Microbacteriaceae*) were selected for and enriched by DTO under DT. Under DC conditions, *Microbacteriaceae* was enriched in both DTO and in DSU, though the enrichment was more pronounced in DTO. Under RF conditions, the *Microbacteriaceae* family made up only 1.1% of DTL and 0.3 of DSL. This phenomenon was mirrored in the cultured microbiome where isolates from genera belonging to *Actinobacteria* represented almost all microbes cultured from drought tolerant wheat lines, including *Curtobacterium flaccumfaciens* (*Microbacteriaceae*) and *Arthrobacter* sp. (*Micrococcaceae*)^12^. The enrichment of *Actinobacteria* in the microbiomes of wheat and other crop species has been consistently observed in multiple drought studies^12,17,26,44,45^. Studies of microbiomes under drought conditions have highlighted the enrichment of *Actinobacteria* by 3.1-fold in wheat as well as a significant increase in agave^12,45^*.* The enrichment of *Actinobacteria* is particularly prevalent in the root microbiome of wheat plants undergoing the early stages of drought stress^12^. While increases in the abundance of *Actinobacteria* has been extensively reported in root microbiomes under drought stress, as far as these authors are aware, this is the first time this trend has been observed in the wheat seed microbiome. It has been proposed that the enrichment of *Actinobacteria* genera under drought could be driven by one or more conserved properties of the *Actinobacterial* lineage^46^.

Seed microbiomes of DTO demonstrated higher levels of *Actinobacteria* enrichment under DT, compared to the seed microbiomes of DSU. Cultivar-specific enrichment of specific genera (and occasionally specific strains) has been demonstrated in potato, common bean, cannabis, sorghum and wheat^47–51^. Wheat cultivars Lewjain, Penawawa and Symphony have been demonstrated to select species of fluorescent pseudomonads from soil microbiomes^52–54^. In root microbiomes, striga-resistant sorghum cultivars enriched for *Acidobacteria* GP1, *Burkholderia, Cupriavidus (Burkholderiaceae), Acidovorax* and *Albidiferax* (*Comamonadaceae*) OTUs, when grown in unfertilised soil^51^. In fact, the relationship between strain and cultivar can be so specific that a bacterial strain that is beneficial in one cultivar can be detrimental in another. For instance in *Brassica napus*, *Paenibacillus polymyxa* Sb3-1 increased growth in the cv. Avata but caused leaf yellowing in the cv. Traviata^33^. In DTO 3 and DTO 4, *Arthrobacter* was enriched 1.5 and 1.9-fold, respectively. This was not observed in drought susceptible lines, suggesting that the enrichment of *Arthrobacter* is dependent on cultivar genetics. Previous studies have demonstrated cultivar-specific selection of an *Arthrobacter* OTU in the rhizosphere of wheat cultivar PI561725 and enrichment of the genera in the presence of 2,4-diacetylphloroglucinol producing species^50^. This study suggests that drought tolerant lines select and enrich for *Actinobacteria* genera when they recruit the seed microbiome for the next generation.

It is worth noting that an increase in diversity under drought could be due to the depletion of dominant taxa, such as *Gammaproteobacteria*, allowing a greater number of OTUs with low abundance to be captured by sequencing. Previous studies of the wheat microbiome under drought stress have not observed the marked depletion in *Gammaproteobacteria* observed across wheat lines in this study. In a study by Naylor et al., there was no detectable change to the abundance of *Gammaproteobacteria* under drought stress^12^. In the profiled seed microbiome, drought stress and wheat lines had significant effects the abundance of *Gammaproteobacteria*. Under drought conditions, all lines showed a marked depletion of *Gammaproteobacteria* families. In DTL, *Enterobacteriaceae* and *Pseudomonadaceae,* the two most dominant families in RC, were significantly depleted under DC. This depletion was not observed to the same extent in DSL under DC. This trend was also observed in the cultured microbiome were genera belonging to *Gammaproteobacteria*, namely, *Pantoea* and *Pseudomonas* dominated the seed microbiomes of drought susceptible lines (DSL), but were depleted in drought tolerant lines (DTL). *Pseudomonas was* depleted significantly in DTL compared to DSL. A similar trend was observed between seed microbiomes under rainfed conditions and drought conditions (DC), where *Pantoea* and *Pseudomonas* were depleted under drought stress. It is possible that the depletion of *Gammaproteobacteria* under drought conditions is a phenomenon that is specific to the seed microbiome*.*

### Seed microbiomes enrich for microbes that promote root growth and drought tolerance

Previous studies have demonstrated that rhizobacteria can be effective in increasing plant biomass and plant survival rate under severe drought conditions in crop plants^27–30^. This study proposes that seed microbiomes may act as a reservoir of PGPB, particularly in DTL under DT. As such seed microbiomes may be a valuable resource for the identification and isolation of PGPB. By introducing single PGPB or an artificial consortium of beneficial bacteria to manipulate the function of seed microbiomes, it may be possible to increasing the productivity and viability of crop species that are subjected to increasingly harsh growing environments. Of the members of the tribe *Triticeae* that were inoculated with representative isolates from the two cultured *Actinobacteria* genera, wheat and oats demonstrated biostimulatory activity. Wheat and oat seeds inoculated with different concentrations of *Cf* D3-25 and *Ar*. sp D4-14 had significantly longer roots, compared to control plants. In wheat, *Cf* D3-25-treated seedlings had an increase in root length between 7.9 and 9.1% and *Ar*. sp D4-14-treated seedlings had a 21% increase in root length. In oat, *Cf* D3-25-treated seedlings solutions had an increase in root length between 63.9 and 104.7% and *Ar*. sp D4-14-treated plants had between 63.9 and 80.1% increase in root length. The inoculation concentration of *Cf* D3-25 did not appear to significantly impact the biostimulatory effect seen in oat and wheat, whereas high concentrations of *Ar*. sp D4-14 inhibited the growth of barley and oats. *Curtobacterium* has been found to associate with roots and promote plant growth in *Arabidopsis*, lettuce, basil, red clover and cucumber^55–57^.

There are a number of microbial traits that are associated with increasing drought tolerance in host plants, specifically the ability to produce 1-aminocyclopropane-1-carboxylate deaminase (ACCd), indole-3-acetic acid (IAA) and siderophores^12^. ACCd regulates ethylene, preventing it from reaching inhibitory levels and thus allowing normal root growth under drought conditions^58^. The auxin analogue IAA enhances shoot and root growth under drought conditions by regulating stomatal aperture^59^. Under drought conditions, siderophore synthesis allows for nutrient cycling^60^. *Cf* D3-25-treated wheat plants showed significant increases in root weight under both mild (26.0%) and severe drought conditions (27.0%), compared to the control. *Cf* D3-25 treated wheat plants also showed a significant increase in wet shoot weight (47.0%) under well-watered conditions, although this effect was less pronounced and not significant under either drought conditions. Both *Cf* D3-25 and *Ar*. sp D4-14 had significantly more fully expanded leaves than the control under well-watered conditions, showing an increase of 35.0% and 27.0%, respectively.

Previous studies of *Curtobacterium* and *Arthrobacter* species suggest that they exhibit PGP activity and traits that can increase drought tolerant of a host plant^62–64^. In lettuce grown under drought conditions, *Curtobacterium herbarum* strain CAH5 increased shoot length, root length and wet biomass by 1.54, 1.23 and 3.84-fold, respectively, compared to control plants^61^. *C. herbarum* CAH5 demonstrated the ability to produce IAA, ACCd, siderophores and solubilise phosphate^61^. *C. flaccumfaciens* colonizes the xylem system of host plants, where it can be either endophytic or pathogenic (causing vascular wilt in bean)^62^. It has been suggested that the prominence of Actinobacteria in plant microbiomes under drought conditions could also be due to where they localise within the plant^62^. Colonisation by xylem-limited bacteria, including Actinobacteria (e.g. *Curtobacterium*), increases resistance to water flow, causing stomatal closure and lowering transpiration^63^. In essence, this phenotype leads to less water loss from the plant, which would be advantageous under drought conditions. Inoculation of *A. nitroguajacolicus* into wheat increased the total dry weight by more than 200% in conditions of high salinity, a condition that often occurs in tandem with drought^64^. *A. nitroguajacolicus* demonstrated the ability to produce auxin, ACCd, siderophores and solubilize phosphate^64^. A*r*. sp D4-14-treated wheat plants did not show a clear drought response in the wheat cv. Bob White. However, as the action of these isolates may be line-specific, it may be prudent to investigate the effects of both isolates on other wheat lines under drought conditions.*Curtobacterium* and *Arthrobacter* isolates are promising candidates for commercial application to increased drought tolerance under a range of conditions.

## Final remarks

This study indicates seed microbiomes from genetically distinct wheat lines enrich for beneficial bacteria in ways that are both line-specific and responsive to environmental stress. As such, they represent an invaluable resource for the further identification of beneficial microbes with plant growth promoting activity and hence improving commercial crop production. Drought stress and plant host genetics affected the composition of the wheat seed microbiome. In turn, drought tolerant lines selected for and enriched certain microbial genera when exposed to either rainfed or drought conditions. Both *Cf* D3-25 and *Ar*. sp D4-14 cultured microbes belonging to taxa enriched by drought tolerant lines under drought conditions, demonstrated the ability to promote plant growth and *Cf* D3-25 increased the growth of wheat under drought conditions. Microbiome profiling suggested the enrichment of microbes was line-specific, therefore, it would be worthwhile to assay key microbes in other drought tolerant wheat lines. Combinations of genetically tolerant and susceptible lines under different stresses could be exploited in a similar manner to find other useful bacteria (e.g. improve nutrient use efficiency).

## Supplementary Information

Supplementary Information.
