# Supplementary material for: Profiling, isolation and characterisation of beneficial microbes from the seed microbiomes of drought tolerant wheat
Source: Sci Rep. 2021 Jun 7;11:11916. doi: 10.1038/s41598-021-91351-8 (PMC8184954; doi:10.1038/s41598-021-91351-8)

Profiling, Isolation and Characterisation of Beneficial Microbes from the Seed Microbiomes of Drought Tolerant Wheat

Holly Hone*[1], Ross Mann[1], Guodong Yang[1,3], Jatinder Kaur[1], Ian Tannenbaum[1,2], Tongda Li[1,2], German Spangenberg[1,2] and Timothy Sawbridge[1,2]

[1]Agriculture Victoria, AgriBio, Centre for AgriBioscience, Bundoora, VIC, Australia.

[2]School of Applied Systems Biology, La Trobe University, Bundoora, VIC, Australia.

[3]College of Animal Science and Technology, Henan University of Science and Technology, Luoyang, Henan, People's Republic of China

Supplementary Figure 1. Phylogram of isolates of *Curtobacterium* species based on MALDI-TOF protein spectra

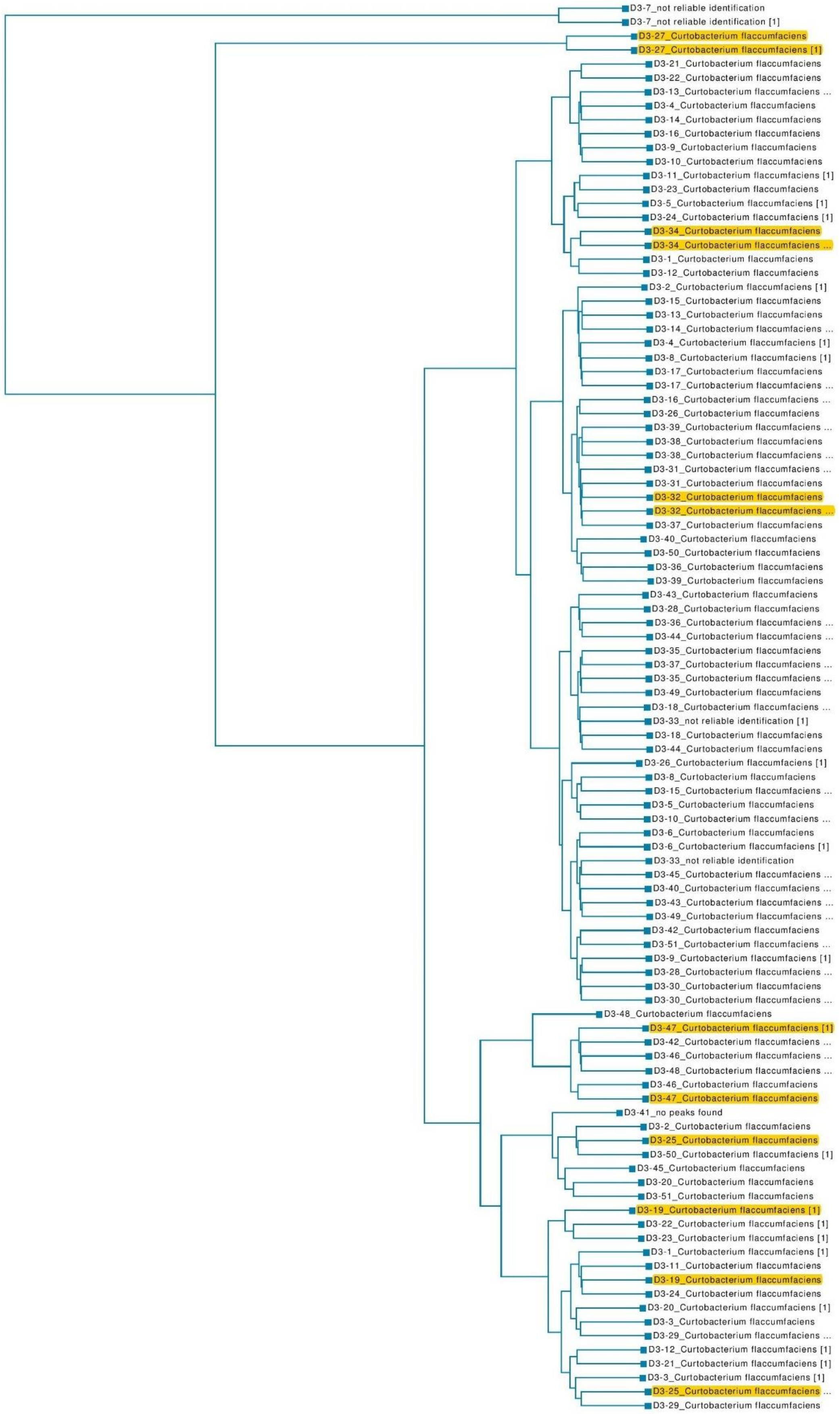

Supplementary Figure 2. Phylogram of isolates of *Arthrobacter* species based on MALDI-TOF protein spectra

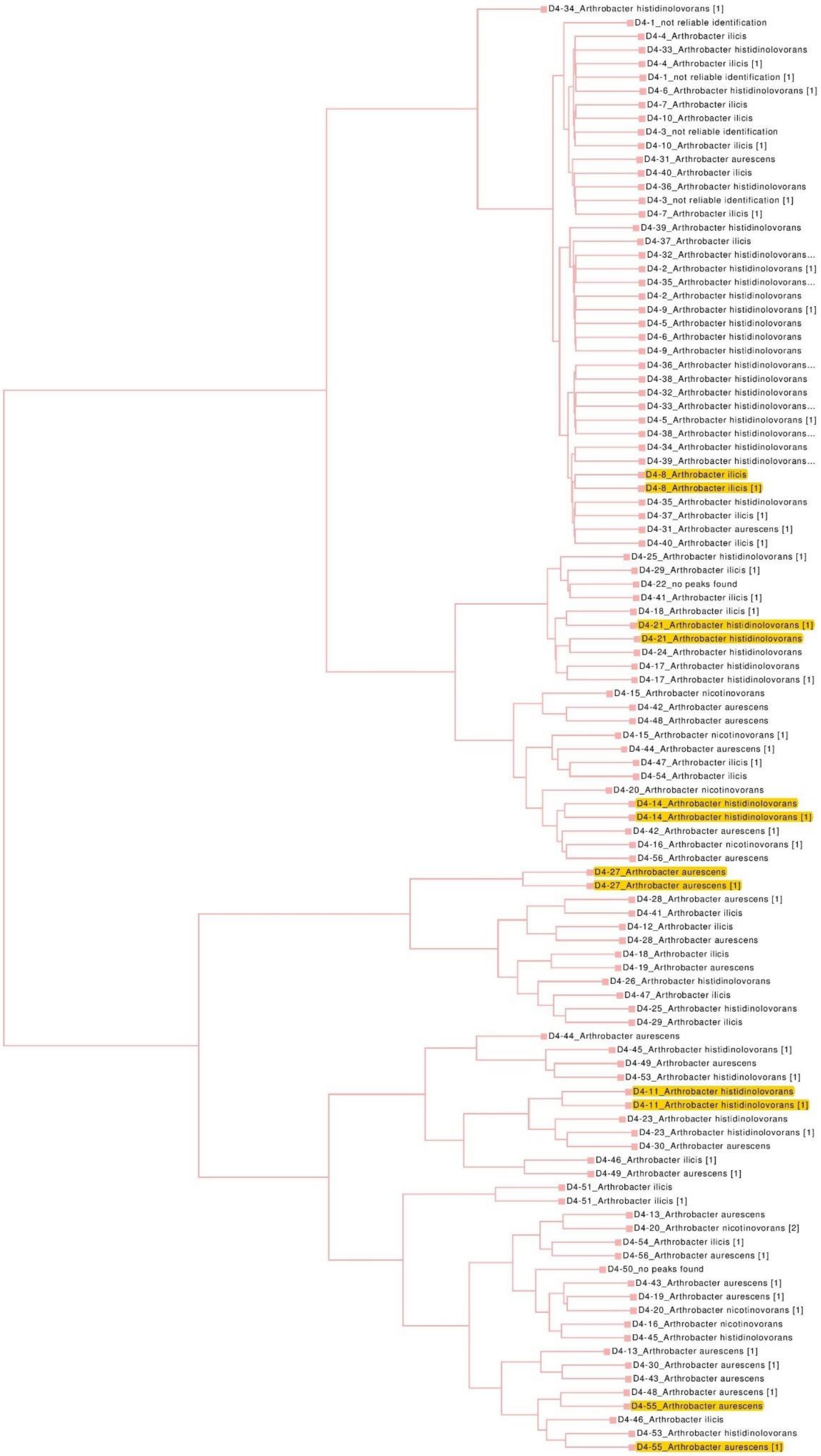

Supplementary Table 1. Pedigree of examined crops

Crop	VASC_ID	Drought Susceptibility Index	Source	Line name	Origin	Status
**Wheat**	DAS5_001935, Line 1	Tolerant	World Wide	Tunis23	Tunisia	Land race
	DAS5_001496, Line 2	Tolerant	World Wide	IDO629	USA	Cultivar
	DAS5_005489, Line 3	Tolerant	Company breeding line			Breeding line
	DAS5_CALINGIRI, Line 4	Tolerant	Flour Samples	Calingiri	Australia	Cultivar
	DAS5_002917, Line 5	Susceptible	AWCC	RAVEN		
	DAS5_003811, Line 6	Susceptible	World Wide	CIGM91.345-5	Mexico	
	DAS5_BRAHAM, Line 7	Susceptible	Flour Samples	Braham	Australia	Cultivar
**Spelt**			Honest To Goodness	ST1040	Australia	
**Oat**			Company breeding line			Breeding line
**Barley**			Seednet	Hindmarsh (formerly VB0324)	Australia	Cultivar
**Ryecorn**			Company breeding line			Breeding line

All experimental research and field trials on plants (including their collection) was conducted in compliance with Victorian, federal, and international guidelines and legislation.

Supplementary Table 2. Genus and number of microbial isolates from Line 3 and Line 4

	Line 3	Line 4
Arthrobacter	0	49
Clavibacter	0	0
Curtobacterium	49	0
Erwinia	0	0
Rathayibacter	0	0
Paenibacillus	0	0
Pantoea	0	0
Pseudomonas	0	0
No identification	2	7
**Total**	51	56

Supplementary Table 3. ANOVA analysis identified the 60 most significant OTUs when comparing microbiomes under drought conditions and microbiomes under rainfed conditions.

OTU number.	OTU ID	Genera	P-value	BH Q-value
1	9af3467db68cf6063627304cecd46a65	*Pseudomonas**	9.04E-18	2.30E-13
2	7ff346973a282aa55de296afdb5d74af	Unknown	2.11E-15	2.69E-11
3	945f562bda86790338922e12f9854407	*Flavobacterium*	7.74E-13	6.57E-09
4	8735f92a7f18db944bc86277fbd3222	Unknown	1.72E-12	1.10E-08
5	fcd80f0cf279eff3c50b66a78d98beeb	*Amycolatopsis*	2.99E-12	1.52E-08
6	8599524cec26e00cc7df72e7ecc01220	Unknown	1.57E-11	6.64E-08
7	d829bee4984f82ffc2453212157caf96	*Bradyrhizobium*	3.68E-11	1.68E-07
8	cc761daf51f27c453da57f3f1f0ff5cc	*Pantoea**	5.80E-11	1.68E-07
9	0a023b056a85b92a1ca8214e78a9c45a	*Skermanella*	5.96E-11	1.89E-07
10	8154c989085dcab535c58fd5a7a18220	*Rubrobacter*	7.45E-11	1.89E-07
11	a869b422007c6d75482b0e9e0338b95c	Unknown	1.64E-10	3.79E-07
12	5f8db894a571d35c9d2fa4f8df8581f9	*Massilia*	2.16E-10	4.59E-07
13	5afec052157f7768bf459883f118a638	*Flavobacterium*	4.25E-10	8.32E-07
14	6abc517aa40e9e7b9c652902fe04bb1a	Unknown	4.84E-10	8.80E-07
15	20425d99034cf4044217704b16934e27	*Noviherbaspirillum*	5.63E-10	9.54E-07
16	889f1bc31887efea2354ba099342a203	*Streptomyces*	6.36E-10	1.01E-06
17	d40c63c872b5cfe4bb4caa9738d10c1f	Uncultured	3.81E-09	5.49E-06
18	35d20da789dde8d76e4389fd6d30cc9d	*Dactylosporangium*	3.10E-09	5.49E-06
19	09f944acc15518b31e5803e1e4cb4e43	*Duganella*	4.35E-09	5.49E-06
20	432b565bc288a18487cfa84b311b7469	Unknown	4.47E-09	5.49E-06
21	205e9006842d47d1a2d1caf6a96e0c88	*Microvirga*	4.53E-09	5.49E-06
22	f6c528c217081be9871de0fc58b89408	*Variovorax*	5.66E-09	6.54E-06
23	6e4b50a611f333bfb12f3b9a4a9e8b61	Uncultured	1.09E-08	1.20E-05
24	f37fc1dccfeba0004cbeb4f1ad6083c6	*Duganella*	1.68E-08	1.78E-05
25	120eba657e42a11a5c29f97b90f02035	*Streptomyces*	2.13E-08	2.17E-05
26	3a66a38841ba6877568198b9dac3a1dc	*Lysobacter*	2.34E-08	2.29E-05
27	ef0c7928901deab56b8d8abef337ac5c	*Rubrobacter*	2.95E-08	2.78E-05
28	cc34ab07649c94630cbc697762ac4b32	*Candidatus*	4.14E-08	3.76E-05
29	79029fa983ec4eda60117a273a52cfc0	*Caulobacter*	4.47E-08	3.92E-05
30	0f8ac0d81dd6449a43b34fcb184efd8f	*Candidatus*	4.86E-08	4.12E-05
31	cb5b9907447a49154182dee3ea78d16e	*Acidibacter*	5.96E-08	4.89E-05
32	0f18144d308ada95632ab5193d92073f	*Pseudomonas**	6.77E-08	5.38E-05
33	bf321c05d79f766c0099afb9ecc42f52	*Streptomyces*	1.21E-07	9.32E-05
34	b712f6d712748de96eb148834a623ba1	*Geodermatophilus*	1.91E-07	1.43E-04
35	a285136eb52ed887013aaa7917ce5051	RB41	3.79E-07	2.75E-04
36	bc3676c17839094c4fa8e33268905268	*Candidatus*	4.40E-07	3.11E-04
37	e08272633711f2ea424a3232ed337e8	Unknown	1.38E-06	9.45E-04
38	1d145835e7f0188562322f4eefb02657	Uncultured	1.72E-06	1.15E-03

39	bc23d6db3bfb2c79f5eb1cec8f01f841	Uncultured	1.86E-06	1.21E-03
40	6e1575cfc367c39ec52c4d1e699a1fb2	*Cellvibrio*	2.14E-06	1.21E-03
41	132ffe9a542685a423a59236950b46b0	Unknown	2.27E-06	1.36E-03
42	bb740c10ad31d5779e628bccffdb5015	*Skermanella*	2.30E-06	1.40E-03
43	e8246466fe381d8a8d5c10cb90640eb3	*Sorangium*	2.49E-06	1.48E-03
44	9b4be26e7bb3a23d25e080a3bba619f6	*Arthrobacter**	2.72E-06	1.57E-03
45	d5620170bb86a5572c5c1b92cd915f05	Uncultured	2.93E-06	1.66E-03
46	00337a2c0aa8af2c100ebca3424b94a1	Uncultured	4.37E-06	2.42E-03
47	a08ee4a8aa1ece798d78fae2b9ad0401	Uncultured	5.24E-06	2.83E-03
48	278a455d0edc27f3fcfb16ad5243ce26	Unknown	5.62E-06	2.98E-03
49	b8c4ed03d7d91546b9fad80b80135a3	*Blastococcus*	6.64E-06	3.45E-03
50	44e2ab81e5e7cff6d965651c707b9479	Uncultured	7.92E-06	4.03E-03
51	caa68010e3e574c4f1069b0ea4e6437e	Blyi10	8.72E-06	4.31E-03
52	fbcfe878584c87b171eafb171b8a0fef	*Rubrobacter*	8.83E-06	4.31E-03
53	1c2b8892a0d7f828d72808cd01cfd199	*Devosia*	8.98E-06	4.31E-03
54	8177ba9a29c738a90c530f54f4561cb1	Unknown	9.37E-06	4.41E-03
55	0fa9b44d52836d2f173f4ac532f8a013	*Skermanella*	1.03E-05	4.75E-03
56	a9164d01308501b57afa0625a15b0f	*Allorhizobium*	1.36E-05	6.16E-03
57	d99a0b2472cd7caecdd8d63ac9cb3fdf	*Rubrobacter*	1.38E-05	6.16E-03
58	26005dfb7ad2e8cdbb139846af329663	*Bacillus*	1.41E-05	6.19E-03
59	c222885a993cabb139b7d8744e6ff7fc	*Labrys*	1.50E-05	6.39E-03
60	ef98cc2984cd3ce0ca6a46589a6df194	Unknown	1.51E-05	6.39E-03

*genus was isolated from wheat seeds

Supplementary Table 4. ANOVA analysis identified the 60 most significant OTUs when comparing microbiomes of drought tolerant lines and microbiomes of drought susceptible lines.

OTU number.	OTU ID	Genera	P-value	BH Q-value
1	2e94aaded5896273b56726409d750804	Unknown	1.50E-11	3.81E-07
2	9c262c0fed9522982fd2297202ee5a83	Uncultured	4.61E-10	5.86E-06
3	aebd4d0ae6384594a5882a958796ded	Uncultured	5.09E-08	0.0004317
4	f1be300df01babcf1897e6103507179f	Uncultured	1.08E-07	0.0006843
5	8b49e37e3c87b34b3cf8fa98e87b9e56	Unknown	6.76E-07	0.003438
6	d2e8afc64cc3c6dde961a4db0a44d26e	*Gemmatimonas*	8.68E-07	0.00368
7	111e8e48a5111d4360329eef12acd3b7	Uncultured	1.87E-06	0.006801
8	ce7ee6ce5f9dd8b3191a0fecd7745de4	Unknown	2.14E-06	0.006805
9	d6bda8545c415bbc3cffa95f43e51e5f	Uncultured	5.41E-06	0.01529
10	cc2a396f24d8239e540d765b422b04eb	Uncultured	6.29E-06	0.01599
11	568bee5fcaa9b6912463e88ef964f3e9	Uncultured	7.11E-06	0.01644
12	8795f8559d860648a3ccc7fe26f49283	Uncultured	8.89E-06	0.01885
13	320fc5aa1aec10458407aedf8f39698f	Unknown	9.73E-06	0.01904
14	02b19fa4ce6a9bd677693ab7e4fceea7	Uncultured	1.20E-05	0.02174
15	d9fe8350aa00c86b865d2f3e18ab18cd	Unknown	1.59E-05	0.02693
16	1c29266ba1c7bc49e8c16e1d5b773041	Uncultured	2.96E-05	0.04697
17	a86ecef5ef052958cda9d725ee257cae	*Gemmatimonas*	3.56E-05	0.05243
18	3e12c438e2563f99c446b789e0d13525	*Gemmatirosa*	3.71E-05	0.05243
19	d1a113d32ccfe6f0c510a20b40a45d91	*Gemmatimonas*	4.02E-05	0.05377
20	7177a92fed62adf72cc159802ac88113	*Gemmatirosa*	5.70E-05	0.07248
21	07f7a53f8a49890a6938be8e5f997f42	*Gemmatimonas*	6.01E-05	0.07283
22	81cf26ae410f4dc2c3782b60aeda6bad	Uncultured	8.38E-05	0.09685
23	097ced6193211c4c49697375f9c0b5e5	Uncultured	9.62E-05	0.1063
24	3576c76306e73ff487d82960da64c73d	Uncultured	1.09E-04	0.116
25	71864b88007f7d9b132ea92e97e7a090	Uncultured	2.97E-04	0.3025
26	710cbf014a0c8791cfc0c6262e928b04	Unknown	3.62E-04	0.3544
27	56841717d2ee29838c93a714e6967889	Uncultured	4.00E-04	0.3771
28	c8e6b05e1033d85877e0b41d1a575b9c	*Gemmatimonas*	4.64E-04	0.4211
29	f34b8c1235d47d75f97eb9680f0a5e4c	Unknown	5.34E-04	0.468
30	1e542d2112237ddb072880742ade6180	*Gemmata*	5.57E-04	0.4725
31	eeb52cc84b4e2c5cea79df01c6787481	*Jahnella*	6.00E-04	0.4923
32	6d987a16ec34bb6be3b91dc00febd060	Ellin6055	7.09E-04	0.5411
33	13bb87c1dcac1cb19f6d9a1d2eee1518	Unknown	7.54E-04	0.5411
34	77a6d64e7b0ba0a9207d305cbd46dc5e	Uncultured	8.25E-04	0.5411
35	314d1f2b81ed24b612e719d7b6669170	Uncultured	1.09E-03	0.5411
36	920a901a455687d1c52d3db8a988a8da3	Uncultured	1.23E-03	0.5411
37	f42b602657b3a6357196f2bbfae84ea4	Uncultured	1.30E-03	0.5411
38	75f0d252adb751ac1592928a7d45d070	Unknown	1.42E-03	0.5411

39	f85631d4c3ad28c56dbfd5389ae3d656	Unknown	1.45E-03	0.5411
40	ab3abeeb031bdd59155c64c511597f76	Unknown	1.54E-03	0.5411
41	8bffc39805f7a62a7b6f44ed0857f583	*Gemmatirosa*	1.57E-03	0.5411
42	8ce415fd299c36132bf28fce66958a18	Unknown	1.61E-03	0.5411
43	3d254eae6330c3af118ad55d15587525	SH-PL14	1.71E-03	0.5411
44	8076cdbe1ee2b24b7c678c67b3ff8c5f	Uncultured	1.77E-03	0.5411
45	02db0dd35b045af9590bd3f21b7dc533	*Gemmatirosa*	1.83E-03	0.5411
46	81add03374eb9a0ac1ac216c305e3fef	Unknown	1.98E-03	0.5411
47	2cdb2f0813b89875f92c58e216b6a3d2	Uncultured	2.10E-03	0.5411
48	71939f43e866e0c70c95d653f060abef	Uncultured	2.32E-03	0.5411
49	6643beaeb166111252c7c651d7224f58	*Blastocatella*	2.48E-03	0.5411
50	895f12b52aba800883fb938fa3bdcd3	Unknown	2.53E-03	0.5411
51	8cdb149aee5a869c9e7a68b271f0a6cf	*Litorilinea*	2.64E-03	0.5411
52	c1427c78632ef67b22025f95e7c34dd7	*Gemmatirosa*	2.68E-03	0.5411
53	ba7b222723a8230a615cab9eff291e99	Uncultured	2.96E-03	0.5411
54	222415525d7e7a1e93033daceb032922	*Chtoniobacter*	2.99E-01	0.5411
55	e5b8aa4e9ca9b255a6e1e7ea5f0c576e	Uncultured	3.10E-03	0.5411
56	edd59c94a0cb54b3e84c87f42f0e8a6a	Uncultured	3.21E-03	0.5411
57	a53e67cee42c20eed1034edf03df170f	*Mucilaginibacter*	3.25E-03	0.5411
58	497a26126c9e726c768bdec79e8c4e9b	*Curtobacterium**	3.29E-03	0.5411
59	30242ff322d5761fc7c180f70f895606	Uncultured	3.37E-03	0.5411
60	5fb8051249a15e13713bc46335d32c25	Unknown	3.39E-03	0.5411

*genus was isolated from wheat seeds

Supplementary Figure 3. Shoot lengths of wheat, barley, oats, ryecorn and spelt inoculated with *Cf* D3-25 and *Ar* sp. D4-14 to assess their biostimulation effects across a range of Triticeae species.

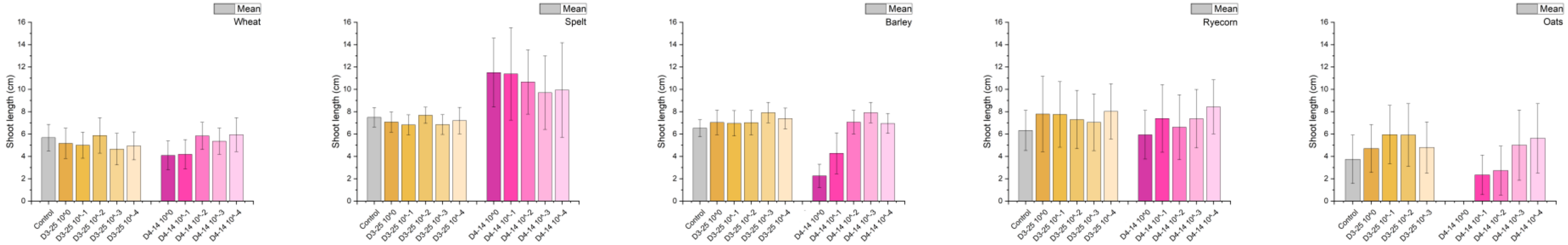

Supplement: Supplementary file 1 — Supplementary Information. [file 41598_2021_91351_MOESM1_ESM.pdf]
